# Time-Series Forecasting Method Based on Hierarchical Spatio-Temporal Attention Mechanism

**DOI:** 10.3390/s25134001

**Published:** 2025-06-26

**Authors:** Zhiguo Xiao, Junli Liu, Xinyao Cao, Ke Wang, Dongni Li, Qian Liu

**Affiliations:** 1 School of Computer Science & Technology, Beijing Institute of Technology, Beijing 100811, China; 3220215169@bit.edu.cn; 2 College of Computer Science and Technology, Changchun University, Changchun 130022, China; 241503554@mails.ccu.edu.cn (J.L.); 241501503@mails.ccu.edu.cn (X.C.); 230702298@mails.ccu.edu.cn (K.W.); 3 National Key Laboratory of Special Vehicle Design and Manufacturing Integration Technology, Baotou 014000, China

**Keywords:** time-series forecasting, BiGRU, SENet, GlobalAttention

## Abstract

In the field of intelligent decision-making, time-series data collected by sensors serves as the core carrier for interaction between the physical and digital worlds. Accurate analysis is the cornerstone of decision-making in critical scenarios, such as industrial monitoring and intelligent transportation. However, the inherent spatio-temporal coupling characteristics and cross-period long-range dependency of sensor data cause traditional time-series prediction methods to face performance bottlenecks in feature decoupling and multi-scale modeling. This study innovatively proposes a Spatio-Temporal Attention-Enhanced Network (TSEBG). Breaking through traditional structural designs, the model employs a Squeeze-and-Excitation Network (SENet) to reconstruct the convolutional layers of the Temporal Convolutional Network (TCN), strengthening the feature expression of key time steps through dynamic channel weight allocation to address the redundancy issue of traditional causal convolutions in local pattern capture. A Bidirectional Gated Recurrent Unit (BiGRU) variant based on a global attention mechanism is designed, leveraging the collaboration between gating units and attention weights to mine cross-period long-distance dependencies and effectively alleviate the gradient disappearance problem of Recurrent Neural Network (RNN-like) models in multi-scale time-series analysis. A hierarchical feature fusion architecture is constructed to achieve multi-dimensional alignment of local spatial and global temporal features. Through residual connections and the dynamic adjustment of attention weights, hierarchical semantic representations are output. Experiments show that TSEBG outperforms current dominant models in time-series single-step prediction tasks in terms of accuracy and performance, with a cross-dataset R^2^ standard deviation of only 3.7%, demonstrating excellent generalization stability. It provides a novel theoretical framework for feature decoupling and multi-scale modeling of complex time-series data.

## 1. Introduction

Driven by the Internet of Things (IoT) and big data technologies, massive amounts of data have been accumulated in fields such as industry, finance, and the natural sciences. Advances in sensor, computing, and communication technologies continuously generate data for time series. These technological innovations have profoundly changed the way complex systems are monitored and controlled. Time-series data, which consists of data points arranged in chronological order, has become a critical element in the operations of many fields. Its continuous accumulation over time and ability to accurately predict future trends play a vital role in practical applications. For example, in the financial sector, the prediction of stock price trends [1] and cash flows directly affects the success or failure of investment decisions. In retail, traffic flow forecasting [2] is used to optimize supply chains and inventory management effectively. Scenarios such as meteorological change analysis and tourism traffic estimation [3] also rely on time-series data to provide solid data support for decision-makers and facilitate scientific decision-making.

The core task of time-series forecasting is to reasonably predict the observed value at time T + 1 based on the data at time T (the current moment). Given the wide applications of time-series forecasting in real life, numerous scholars have conducted in-depth research in this field. Currently, mainstream forecasting methods are mainly divided into traditional forecasting methods and machine learning-based forecasting methods. Traditional methods include the autoregressive (AR) [4], Moving Average (MA) [5], Autoregressive Moving Average (ARMA) [6], and Autoregressive Integrated Moving Average (ARIMA) [7] models, among others. However, these methods still have certain limitations when faced with complex and changeable real-world data. This study aims to overcome the existing challenges in time-series forecasting, further improve the effectiveness and accuracy of forecasting, and provide more reliable technical support for the development of related fields.

In recent years, deep learning has emerged as a new approach in which each module transforms one level of abstraction into another, enabling deep learning to learn features from data. In the field of time-series forecasting, the deep learning model RNN [8] has been widely used; however, it is prone to gradient disappearance and explosion during training. To address this, a Long Short-Term Memory Network (LSTM) [9] and GRU [10] were developed. Although they effectively solved the gradient problem, the prediction accuracy remained unsatisfactory. Compared with traditional recurrent networks, such as LSTM and GRU, TCN [11] has higher accuracy and a simpler structure. However, it does not consider the weight relationship between channels, leading to low-importance information participating in the calculations and weakening the weight of key information. While GRU can handle long-range dependencies and avoid gradient disappearance to a certain extent, its reset gate mechanism tends to ignore most of the current information during processing. Although BiGRU [12] can bidirectionally extract local features, it lacks the ability to integrate global information, making it difficult to achieve dimensionality elevation from local time-domain features to global vision feature representations. Although TCN and BiGRU variant models combined with attention mechanisms have shown certain advantages in time-series forecasting, such models only optimize network performance from a single dimension, lacking multi-scale and multi-level information interaction and fusion, and thus struggle to fully exploit complex features and dependency relationships in the data, which limits further improvements in model performance. To address the long-range dependency problem, models combined with attention mechanisms have become a research hotspot. For example, Attention-LSTM enhances the focus on key time steps through a self-attention mechanism; however, the computational complexity increases quadratically with the sequence length. The Informer uses a sparse attention mechanism to reduce the complexity to linearity, achieving a 2.3-fold improvement in the inference speed for meteorological data prediction. However, such models are mostly optimized from a single dimension (e.g., only enhancing attention in the time dimension) and lack weight screening in the feature dimension. In the field of time-series forecasting, traditional and deep learning models have long been constrained by the dual challenges of single-dimensional feature extraction and insufficient long-range dependency modeling capabilities. This study proposes the TSEBG hybrid network model, which deeply integrates SENet [13] and the Global Attention mechanism [14] into the TCN and BiGRU models. It primarily reconstructs the time-series forecasting paradigm through three core innovations:

(1) This study proposes a TSEBG hybrid network model that innovatively fuses channel and global attention mechanisms. Specifically, the SENet is embedded in the TCN to construct a dynamic weight-allocation system that enhances the cross-dimensional features. Global attention is introduced into BiGRU to build a long-range dependency framework that accurately captures sequential features. The collaboration of these two mechanisms breaks through traditional bottlenecks and significantly improves the prediction accuracy of multivariate time series.
(2) To address the challenge of fragmented processing of local and global information in traditional models, a hierarchical collaborative feature fusion network is constructed. Based on a dual-channel structure, it realizes complementary learning of multi-time-scale features and designs a cross-layer fusion strategy to achieve multi-level alignment of spatial-temporal information. Synergistically optimizing local details and the global context-time series significantly enhances the representation capability of complex time-series data.
(3) The TSEBG model was applied to time-series forecasting and compared with mainstream deep learning models on the Electricity Transformer Temperature (ETT) dataset. The results of the experiments show that the model reduces the root mean square error (RMSE) by an average of 18.4%, decreases the mean absolute error (MAE) by 21.9%, and significantly optimizes R^2^. With its dual-channel collaboration and cross-layer fusion architecture, the TSEBG can capture time-series features more accurately and demonstrate excellent error control capabilities in single-step prediction tasks.

This study focuses on the shortcomings of traditional and deep learning methods in feature extraction and long-range dependency modeling for time-series forecasting and proposes the TSEBG model enhanced by dual attention mechanisms. By innovatively integrating attention mechanisms and constructing a hierarchical feature collaboration network, it breaks through existing technical bottlenecks. Experiments confirmed that the proposed model exhibited strong performance and prediction capabilities. This study provides new ideas and methods for constructing time-series forecasting models and enriches the theoretical system of time-series forecasting.

## 2. Related Work

The development of time-series forecasting models began with the autoregressive (AR) model proposed by the British statistician Yule. The core of this model lies in the fact that the output variable largely depends on its own previous values and random conditions. To enhance predictive capabilities, the academic community has improved the AR model by deriving the Moving Average (MA) and Autoregressive Moving Average (ARMA) models. However, the ARMA model has limitations in handling non-stationary time-series data. To address this issue, the Autoregressive Integrated Moving Average (ARIMA) model was developed, which stabilizes data through one or more differencing steps. However, differencing operations often amplify data noise and affect the prediction accuracy. Based on this, Peter and Slivia [15] proposed the Autoregressive Integrated Moving Average with Exogenous Variables (ARIMAX) model, which introduces external variables to enhance the explanatory power of time-series data.

Traditional time-series forecasting methods have obvious shortcomings. On the one hand, they have large prediction errors, struggle to uncover dynamic relationships between multiple variables, and fail to effectively capture complex dependency structures. On the other hand, they exhibit low efficiency and high computational complexity when processing large-scale datasets. To overcome these drawbacks, machine learning techniques have been widely applied to non-stationary time-series forecasting, such as support vector machines (SVM) and neural networks [16]. Thissen et al. [17] and Gui et al. [18] proposed a prediction model based on support vector machines (SVM) called support vector regression (SVR), which yields better results than ARIMA. Least squares support vector machines (LS-SVM) modify the constraints of the objective function. Wang and Hu [19] compared SVM and LS-SVM in solving regression prediction problems and found that LS-SVM is more suitable for large-scale problems. These methods can automatically learn features from data and have stronger modeling capabilities for complex, nonlinear relationships. Sun and Zhao [20] compared the short- and long-term performance differences between artificial neural networks (ANN) and LS-SVM in exchange rate forecasting, verifying the advantages of LS-SVM in handling large-scale data and complex nonlinear relationships. Zhao and Xia [21] applied neural networks to short-term time-series forecasting of nonlinear data.

In recent years, deep learning has been applied to time-series forecasting. Wu and Xu et al. [22] proposed the Autoformer model, which combines sequence decomposition and autocorrelation mechanisms to significantly improve long-term time-series forecasting performance; Liu et al. [23] proposed innovative solutions for the non-stationarity of time series. Kaleem and Muhammad [24] proposed a hybrid model combining CNN and LSTM to predict load values at the next time point under STLF (Short-Term Load Forecasting), demonstrating significantly improved performance compared to the traditional ARIMA model. Dasgupta and Osogami [25] proposed a Gaussian Dynamic Boltzmann Machine (Gaussian DyBM) by integrating an RNN. The parameter update method of the Gaussian DyBM does not rely on backpropagation algorithms; therefore, parameter updates are independent of data dimensions or maximum lags. These features render the Gaussian DyBM more robust and scalable. In 2023, WEN et al. [26] proposed an LSTM-Attention-LSTM parallel prediction model and compared it with some CNN and LSTM variant models. In the same year, Cai et al. [27] proposed a method based on the parallel fusion of CNN and GRU to improve the accuracy and efficiency of power-quality disturbance classification. However, LSTM has black-box properties, making its internal operating mechanisms difficult to intuitively interpret and leading to insufficient interpretability. RNN [28] can set output data based on current and past input sequences, but they use the Backpropagation Through Time (BPTT) algorithm for network training, which causes gradient disappearance when processing long time intervals sequentially. GRU also has limitations in interpretability, making it difficult to clearly demonstrate decision-making processes and logically understand data features. To address these issues, this paper designs a model that combines a Temporal Convolutional Network (TCN) with a Bidirectional Gated Recurrent Unit (BiGRU), endowing it with strong capabilities to effectively capture sequence patterns and dependency relationships between elements. Therefore, channel and global attention mechanisms were introduced to further enhance the model’s precise time-series modeling capabilities, especially in handling complex seasonal and periodic patterns. This allows the model to focus more accurately on key information and achieve accurate analysis and processing of time series.

In summary, during the iterative development of time-series forecasting models from traditional to machine learning and deep learning models, while there have been certain improvements in prediction accuracy, prediction errors have not been significantly reduced. This paper innovatively proposes a TSEBG time-series forecasting model. To address issues such as the non-stationarity of time series, we propose starting with strengthening sequence feature capture and key information, designing a multimodal feature fusion network to model sequences from different perspectives, and achieving high-precision time series forecasting.

## 3. Methodology

**Figure 1:**
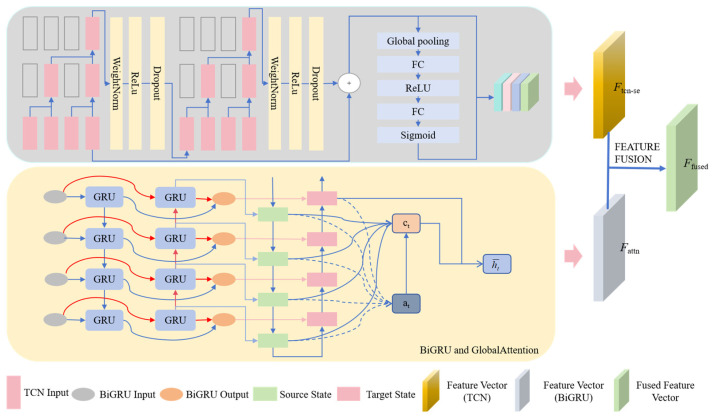
TSEBG model structure diagram.

To address the challenges of feature extraction and dependency modeling in time-series forecasting, the TSEBG model proposes a dual-channel collaborative feature extraction and spatio-temporal feature fusion network architecture. This architecture fully integrates the advantages of the TCN and BiGRU. The dual-domain attention collaborative network achieves adaptive weight allocation for the Temporal Convolutional Network (TCN) and integrates the global time-domain features extracted by the Bidirectional Gated Recurrent Unit (BiGRU), enabling the efficient decomposition of multi-scale features and cross-layer fusion. This allows the model to accurately capture long- and short-term dependencies, strengthen key feature representation, and overcome the single-dimensional processing limitations of traditional models. The detailed architecture is illustrated in Figure 1.

### 3.1. Spatio-Temporal Convolutional Feature Adaptive Extraction Network Based on SENet

**Figure 2:**
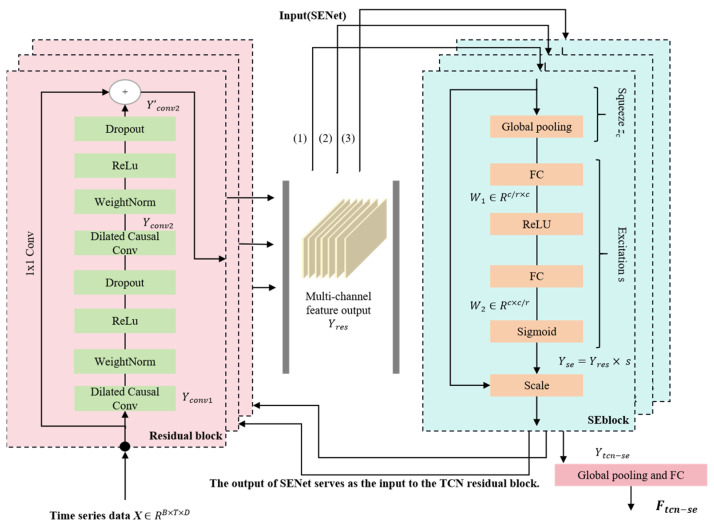
TCN Network structure diagram integrated with SENet.

To overcome the limitations of traditional Temporal Convolutional Networks (TCN) in single-dimensional feature processing, the TSEBG model proposed in this paper constructs a first-of-its-kind feature extraction channel. By innovatively integrating the channel attention mechanism (SENet) with the Temporal Convolutional Network (TCN), it achieves adaptive adjustment of the channel weights in the TCN network. This enables cross-dimensional dynamic enhancement and selective extraction of time series features, effectively addressing the single-dimensional feature processing limitations of traditional TCN. Its multi-scale information mining capability lays the foundation for subsequent multimodal feature fusion and long-range dependency modeling. The detailed network architecture is illustrated in Figure 2.

In this channel, the input data $X\in R^{B\times T\times D}$ is received, where B represents the batch size, T is the number of time steps, and  D is the feature dimension. The output $Y_{conv1}$ of the dilated causal convolution of the first residual block in TCN is:

(1) $$Y_{conv1}=\sum_{k=0}^{K-1}w_{k}\times X_{t-d\cdot k}$$

where $w_{k}$ represents the weight of the convolution kernel, d is the dilation factor, whose value grows exponentially with the increase of network depth; K represents the number of convolution filter windows (kernels), and k refers to a specific convolution filter window. This operation can effectively expand the receptive field without increasing the computational load and only considers data from the current and past time steps without involving future data [29]. Subsequently, it passes through normalization, the activation function $\left(ReLU(Y_{conv1})\right)$ and the Dropout layer in sequence. Since the residual block of TCN contains this process twice, the output of the second—layer dilated causal convolution is $Y_{conv2}$, which then passes through normalization, the activation function, and the Dropout layer again, and the final output is $Y_{conv2}^{\prime }$. Finally, a residual connection operation is performed within the residual [30] block as follows:

(2) Yres=ReLUYconv2′+Conv1D1×1(Y)

In this process, there is a 1D convolution operation to ensure that X and $Y_{conv2}^{\prime }$ can have aligned dimensions for element—wise addition. This connection method not only helps stabilize the training process but also enables the capture of richer information (Yang et al. [31]). The output $Y_{res}$ of the TCN residual block is a multi—information channel. To adaptively select the most discriminative subset of information, we introduce the SENet channel attention mechanism here. Taking $Y_{res}$ as the input of SENet, the global average pooling (Squeeze) operation is performed:

(3) $$z_{c}=\frac{1}{T}\sum_{t=1}^{T}Y_{res,t}$$

$z_{c}$ is the compressed value of the c-th channel. This operation aggregates the global information of the feature maps. By compressing the feature map in the spatial dimension, the two-dimensional spatial information of each feature map channel is transformed into a real number, thus obtaining a channel descriptor. This descriptor can capture the global statistical information of the entire feature map across all the channels. Subsequently, an excitation operation is performed. The channel weights are learned through two fully—connected layers:

(4) s=σW2⋅ReLUW1⋅z

where $W_{1}\in R^{C/r\times C}$ and $W_{2}\in R^{C\times C/r}$ are the weights of the fully connected layers, and the reduction factor is 16. First, this operation is processed by a fully—connected layer, and the output size is (C/reduction); subsequently, it passes through a ReLU activation layer, and then through another fully—connected layer, the dimension is restored to the original C. Finally, sigmoid normalization is performed to obtain a value between 0 and 1, which is the weight of each channel. In the last step of SENet, the weight s is multiplied (scale) with the corresponding channel of the original input $Y_{res}$ to obtain the final output $Y_{se}$:

(5) $$Y_{se}=Y_{res}\cdot s$$

The TCN network of the TSEBG model contains multiple residual blocks. We introduce the SENet attention mechanism after each residual block. The output of the SENet in the first layer is then used as the input for the residual block in the second layer. Through this alternating stacking of TCN and SENet, a hierarchical feature extraction structure is formed:

(6) $$Y_{tcn-se}=SEBlock(TCN(SEBlock(TCN(X))))$$

Finally, through global average pooling and a fully—connected layer, the prediction result $F_{tcn-se}$ is generated. In the model architecture, the SENet and TCN modules form a cascaded processing unit of “temporal convolutional feature extraction—channel attention optimization”. This not only provides a more focused feature input for the subsequent temporal dynamic modeling of BiGRU but also significantly enhances the model’s ability to represent complex time—series patterns through the re-distribution of information in the channel dimension.

### 3.2. Bidirectional Gated Global Temporal Feature Extraction Network Based on Global Attention Mechanism

**Figure 3:**
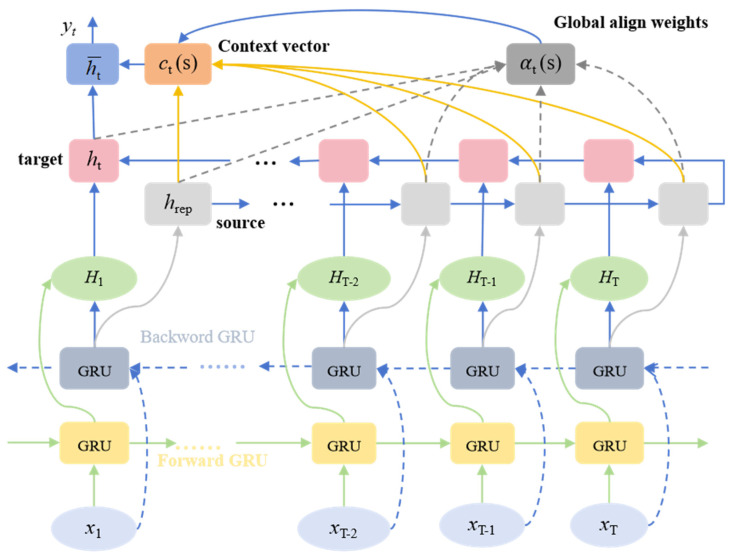
Flowchart of the BiGRU model with the introduction of GlobalAttention.

To achieve in-depth modeling of complex dynamic features in time-series data, this paper innovatively constructs a Bidirectional Gated Recurrent Unit (BiGRU) based on a global attention mechanism as the second feature extraction channel. This module breaks the traditional unidirectional information processing model and deeply couples the bidirectional information flow structure with the global attention mechanism to build a novel architecture with spatio-temporal feature perception. This module can simultaneously capture long-range dependencies in time-series data from both forward and reverse dimensions, accurately screen, and effectively integrate temporal context information in the sequence. The detailed network architecture is illustrated in Figure 3.

In the TSEBG model, BiGRU and TCN jointly receive the input sequence $X\in R^{B\times T\times D}$, where B represents the batch size, T is the number of time steps, and D is the feature dimension. BiGRU ingeniously concatenates two GRU modules. Its mechanism is rooted in splitting standard GRU neurons into two different states: forward and backward (representing positive and negative time directions, respectively) [32]. X is first output through forward and backward GRUs. The GRU is composed of two key gate structures, the update gate *z_t_* and the reset gate *r_t_*, and the calculation formulas are as follows:

(7) $$z_{t}=\sigma \left(W^{z}\cdot x_{t}+U^{z}\cdot h_{t-1}\right)$$

(8) $$r_{t}=\sigma \left(W^{r}\cdot x_{t}+U^{r}h_{t-1}+b^{r}\right)$$

(9) $${h^{\prime }}_{t}=\tanh\left(W^{h^{\prime }}\cdot x_{t}+U^{h^{\prime }}\cdot \left(r_{t}⊙h_{t-1}\right)+b^{h^{\prime }}\right)$$

(10) $$h_{t}=\left(1-z_{t}\right)\times h_{t-1}+z_{t}\times h_{t}^{\prime }$$

For a single time step t, the input is $x_{t}\in R^{B\times D}$, and the hidden state at the previous time step is $h_{t-1}\in R^{B\times H}$ (H is the dimension of the hidden layer). In this process, the combination of the new input $x_{t}$ and the hidden state of the previous time step (which can be understood as the previous memory) $h_{t-1}$ is adjusted by the reset gate $r_{t}$. $r_{t}$ can control the degree to which the information of the previous hidden state is retained at the current time step, thus flexibly determining how much historical information participates in the current calculation. The update gate $z_{t}$, on the other hand, is responsible for controlling the retention of the previous hidden state $h_{t-1}$, thereby determining the degree to which the new hidden state $h_{t}$ inherits historical information.

The forward GRU unit processes the input sequence to compute the forward hidden state sequence $h_{t}^{fwd}$ for all time steps, capturing the contextual information from the past to the future. Concurrently, the backward GRU processes the reversed input sequence to generate the backward hidden state sequence $h_{t}^{bwd}$, capturing the contextual information from the future to the past. Subsequently, the bidirectional hidden states at each time step t are concatenated as follows:

(11) $$H_{t}=\left[h_{t}^{fwd};h_{t}^{bwd}\right]$$

Finally, the output of BiGRU is the bidirectional hidden states for all time steps $H=[H_{1},H_{2},\ldots ,H_{T}]\in R^{B\times T\times 2H}$. In the TSEBG model, the number of BiGRU layers is greater than one. To calculate the importance weights for each time step and perform weighted aggregation on the hidden state sequence H output by BiGRU, focusing on key temporal features, we introduce a global attention mechanism after BiGRU. Taking the output H of the last BiGRU layer as the target value, and the final hidden state $h_{final}\in R^{B\times H}$ of the last backward GRU in the last BiGRU layer as the source state (i.e., the query vector). Here, we repeat $h_{final}$ T times along the time step dimension to obtain $h_{rep}\in R^{B\times T\times 2H}$, and then concatenate H and $h_{rep}$ to obtain $h_{con}\in R^{B\times T\times 4H}$. The energy vector is calculated using a linear transformation as follows:

(12) $$e_{t}=\tanh\left(W_{attn}h_{con}+b_{attn}\right)$$

where $W_{attn}\in R^{2H\times 4H}$ and $e_{t}\in R^{B\times T\times 2H}$. The weight vector is calculated using this energy vector $e_{t}$ as follows:

(13) $$\alpha_{t}=\mathrm{soft}\max\left(w_{v}^{T}e_{t}\right)$$

Here, $w_{v}\in R^{2H}$, $\alpha_{t}\in R^{B\times T}$, and the sum of the weight vectors across all time steps is 1. This weight vector $\alpha_{t}$ reflects the degree of importance of each source state for the current target state. Finally, the global context vector is generated as follows:

(14) $$c=\sum_{t=1}^{T}\alpha_{t}\times H_{t}$$

When deriving the context vector $c_{t}$, all the hidden states of $H_{t}$ are comprehensively considered. Thus, the final output $F_{attn}$ of this channel is obtained. This process not only effectively focuses on the sequence segments that are most valuable for the current task, but also, through the integration of information across time steps, elevates the local temporal features extracted by the BiGRU to a global feature representation.

### 3.3. Local Spatial and Global Temporal Feature Fusion Channel

In the final stage of model construction, a dynamic gating feature fusion strategy is adopted. Using learnable gating weights, the contribution ratios of the TCN and BiGRU features are adjusted in real-time, addressing the limitations of static weights in traditional feature fusion methods.

In this module, first, we map the final output features $F_{tcn-se}$ and $F_{attn}$ of the two channels to the same dimension D=embed_dim through linear transformations. The calculation formula is as follows:

(15) $$TSTransformed=W_{tcn}\times F_{tcn-se}+b_{tcn}$$

(16) $$BGTransformed=W_{gru}\times F_{attn}+b_{gru}$$

The output dimensions are $TSTransformed\in R^{B\times D}$ and $BGTransformed\in R^{B\times D}$. Then, through a gating mechanism, the original features are concatenated as $gate=Concat(F_{tcn-se},F_{attn})$ to generate a gating signal, which is used to adaptively determine the importance of the two types of features as follows:

(17) $$gatevalue=\sigma (W_{g}\times gate+b_{g})$$

where $W_{g}\in R^{1\times (C_{tcn}+C_{gru})}$ is the weight of the linear layer, $b_{g}\in R^{1}$ is the bias, σ is the Sigmoid function, which compresses the gate value to the range of 0 to 1, and the output dimension is $gatevalue\in R^{B\times 1}$. Subsequently, the two types of features are weighted and summed using the gate value as follows:

(18) $$F_{fused}=gatevalue\times TSTransformed+(1-gatevalue)\times BGTransformed$$

When gatevalue≈1, the fusion result is dominated by the TCN—SENet features; when gatevalue≈0, the fusion result is dominated by the BiGRU—GlobalAttention features; intermediate values indicate the mixing ratio of the two types of features, which is dynamically determined by the input data.

Finally, the fused features are mapped to the prediction dimension using a linear layer:

(19) $$predict=W_{fc}\times F_{fused}+b_{fc}$$

This feature fusion method achieves efficient fusion of temporal—spatial features and temporal—context features through mathematical linear transformations and non—linear activations, combined with dynamic weight allocation, providing a more discriminative feature representation for subsequent prediction tasks.

The dual—channel feature extraction architecture adopted by the TSEBG model: On the one hand, dilated convolutions are used to mine multi—scale temporal dependency features, and combined with SENet to adaptively calibrate channel weights, extracting important spatial features of time series; on the other hand, by dynamically generating attention weights, it focuses on the temporal features relevant to the prediction step, enhancing the ability to capture long—range temporal dependencies. Finally, after dynamic gating feature fusion, the fully—connected layer completes the prediction. The pseudocode for the TSEBG is described in Algorithm 1.
**Algorithm 1** TSEBG
Require: model parameters initialize the model: Model=TSEBG, sliding window
Input: Time-series
$X=(X_{1},X_{2},X_{3},\ldots ,X_{t})$
1While stopping criterion is not satisfied do:2    channel one: temporal-spatial feature extraction3   Dilated Caulsal Conv output:
$Y_{conv1,conv2}=\sum_{k=0}^{K-1}w_{k}\times X_{t-d\times k}$
4   WeightNorm, ReLu and Dropout Layer:
${\hat{Y}}_{conv2}$
5   Residual Calculation:
$Y_{res}=RELU({\hat{Y}}_{conv2}+{Conv1D}_{1\times 1}(Y))$
6   Squeeze and Excitation:
$z_{c}=\frac{1}{T}\sum_{t=1}^{T}Y_{res,t}$ 
$s=\sigma (W_{2}\times ReLU(W_{1}\times z))$
7   Scale:
$Y_{se}=Y_{res}\times s$
8   Connect SENet after each TCN residual block to compute channel weights:         $Y_{tcn-se}=SEBlock(TCN(SEBlock(TCN(X))))$
9   After global average pooling and a fully connected layer:
$F_{tcn-se}$
10    channel two: time-domain feature extraction11   The input data passes through the forward and backward GRU units sequentially:12    Update Gate:
$z_{t}=\sigma (W^{z}x_{t}+U^{z}h_{t-1})$
13    Reset Gate:
$r_{t}=\sigma (W^{r}x_{t}+U^{r}h_{t-1}+b^{r})$
14    Candidate State:
$h_{t}^{\prime }=tan(W^{h^{\prime }}x_{t}+U^{h^{\prime }}(r_{t}⊙h_{t-1})+b^{h^{\prime }})$
15    Current Hidden State:
$h_{t}=(1-z_{t})h_{t-1}+z_{t}{h_{t}}^{\prime }$
16   Concatenate the bidirectional hidden states:
$H_{t}=[h_{t}^{fwd};h_{t}^{bwd}]$
17   Concatenate the output of the BiGRU with the final hidden state of the backward layer from the last BiGRU layer, repeated T times, to obtain
$h_{con}$
18   Calculate the energy vector:
$e_{t}=tanh(W_{attn}h_{con}+b_{attn})$
19   Calculate the weight vector:
$\alpha_{t}=softmax(w_{v}^{T}e_{t})$
20   Finally generate the global context vector and ouput
$F_{attn}$: $c=\sum_{t=1}^{T}\alpha_{t}\times H_{t}$
21    Feature Fusion:22   Feature Mapping:
$TSTransformed=W_{tcn}\times F_{tcn-se}+b_{tcn}$          
$BGTransformed=W_{gru}\times F_{attn}+b_{gru}$
23   Concatenate the original features:
$gate=Concat(F_{tcn-se},F_{attn})$
24   Generate a gating signal:
$gatevalue=\sigma (W_{g}\times gate+b_{g})$
25    Use the gating value to perform a weighted summation of the two types of features:  fused=gatevalue×TSTransformed+(1−gatevalue)×BGTransformed
26    The fused features are mapped to the prediction dimension via a linear layer:             $predict=W_{fc}\times fused+b_{fc}$
27end

## 4. Experimental Evaluation

In this section, we first introduce the evaluation metrics and relevant information of the datasets. Subsequently, we describe the process of pre-processing the dataset in detail. In addition, a comprehensive and meticulous introduction to the relevant parameter settings of the experiment is provided, including the rationale and principles for determining the values of key parameters, such as model hyperparameters, number of training epochs, and learning rate. Finally, an in—depth analysis and evaluation of the experimental results will be carried out, interpreting the performance of the model in various evaluation metrics from different dimensions, and exploring the model characteristics, dataset features, and the essence of the research questions reflected behind the experimental results.

### 4.1. Evaluation Indicators

In time-series forecasting research, the selection of evaluation metrics must balance the generality of the methods with their adaptability to specific scenarios. This paper employs three core evaluation metrics widely used in academia: Root Mean Squared Error (RMSE), Mean Absolute Error (MAE), and Coefficient of Determination (R^2^). The mathematical definitions are as follows:

(20) $$RMSE=\sqrt{\frac{1}{n}\sum_{i=1}^{n}{\left(y_{i}-{\hat{y}}_{i}\right)}^{2}}$$

(21) $$MAE=\frac{1}{n}\sum_{i=1}^{n}\left|y_{i}-{\hat{y}}_{i}\right|$$

(22) $$R^{2}=1-\frac{\sum_{i=1}^{n}{\left(y_{i}-{\hat{y}}_{i}\right)}^{2}}{\sum_{i=1}^{n}{\left(y_{i}-\bar{y}\right)}^{2}}$$

In the equations, $y_{i}$ represents the true value of the *i*-th data point in the sequence, ${\hat{y}}_{i}$ is the predicted value of the i-th data point at the corresponding time, $\bar{y}$ is the sample mean of the true values, and n is the sample size of the time series. In terms of the evaluation logic, RMSE amplifies the prediction deviation through the squared term and mainly reflects the prediction accuracy of the model at extreme points. In contrast, MAE measures the overall deviation from the mean of absolute errors, and its physical meaning is more intuitive. The smaller the values of these two metrics, the lower the degree of deviation between the predicted and true values, and the better the prediction ability of the model. As a goodness—of—fit indicator, R^2^ quantifies the ability of the model to explain the variation in data by comparing the prediction error with the benchmark error (the sum of squared deviations between the true values and the mean). Its value range is (−∞,1]. When R^2^ approaches 1, it indicates that the model’s prediction results highly coincide with the true sequence, and the prediction accuracy reaches an ideal state.

### 4.2. Datasets

During this experiment, we used three open—source datasets, namely the oil temperature datasets ETTh1, ETTm2, and ETTh2. Table 1 presents the analytical information of the three datasets. These three datasets provide rich data support for experiments from different perspectives and play a key role in the field of time—series forecasting and analysis.

(1) ETTh1

The ETTh1 dataset is an important part of the Electricity Transformer Temperature (ETT) dataset, which aims to promote research in time—series forecasting. In the development of power systems, accurately predicting the temperature of transformers is crucial for ensuring the stable operation of the system. Focusing on this scenario, ETTh1 encompasses multi—dimensional attributes such as time, oil temperature, load, and weather. It is a typical multivariable time-series dataset, providing strong support for the training and evaluation of multivariable time-series forecasting models.

(2) ETTm2

The ETTm2 dataset focuses on data related to transformers in power systems and presents them in the form of time series. This dataset provides a standard testing platform for researchers and engineers. By conducting various experiments on time—series forecasting models using this dataset, researchers can clearly understand the advantages and disadvantages of different models in handling transformer time—series data and then optimize and improve the models. This strongly promotes the application and development of time—series forecasting techniques in the field of power systems.

(3) ETTh2

The ETTh2 dataset is part of the Electricity Transformer Temperature (ETT) dataset. It consists of oil temperature data of power transformers from two different counties in the same province in China, provided by the State Grid. The data contains historical data from 1 January 2012, to 31 December 2015, with a time span of four years. Each data point contains 8—dimensional features, including the recording date of the data point, the predicted target value “oil temperature”, and six different types of power load features. This dataset is often used in academic research and experiments related to time—series forecasting, such as predicting the oil temperature of power transformers and studying the ultimate load—bearing capacity of power transformers, providing data support for the training and validation of relevant algorithms and models.

(4) Weather

This dataset is a meteorological observation dataset consisting of 21 meteorological parameters at 10 min intervals from the Jena weather station in Germany. The data contains historical data from 1 January 2020 to 1 January 2021, spanning a time period of 1 year.

(5) Exchange_rate

The Exchange Rate dataset is a time-series dataset that covers the daily exchange rate fluctuations of eight countries from 1990 to 2016. This dataset is commonly used for foreign exchange market analysis, economic forecasting, financial modeling, and machine learning time-series prediction.

### 4.3. Experimental Settings

In this study, given the differences in the data collection time cycles between the ETTh and ETTm datasets, the hyperparameter settings during the experiment also varied. Specifically, the batch size was set to 128 for the ETTh1 and ETTh2 datasets, while it was adjusted to 64 for the ETTm2 dataset. To balance the model training effectiveness and efficiency, the number of epochs was set to 100, and an early stopping mechanism with a patience value of 10 was adopted to effectively prevent overfitting. In the TCN module, the step size was consistently set to 1, and the dilation factor was set to $2^{\left(number-1\right)}$ to expand the receptive field and enhance the model’s ability to capture time-series features. The detailed parameters of the TSEBG model for different datasets are listed in Table 2. To accelerate model convergence and mitigate the gradient vanishing problem, the RELU was selected as the activation function. The dimensions of the attention layer were set to be consistent with those of the BiGRU output layer to ensure the effectiveness of information transmission and feature extraction in the model. During the model training phase, the MSE loss function was used to accurately measure the prediction errors, and the Adam optimization algorithm was employed with a learning rate set to 0.0003.

The core differences among these five datasets are focused on time granularity. Specifically, ETTh and exchange_rate are hourly and daily data, respectively, featuring long time cycles and relatively sparse data points; ETTm2 and weather datasets are minute-level data, characterized by short time cycles and denser data points. These differences in data characteristics necessitate the adaptation of models to different time-series feature scales and the handling of varying computational complexities. In the comparative experiments, minor adjustments were made to the model’s parameters. The key parameter adjustments for the comparative models are listed in Table A1.

### 4.4. Data Pre-Processing

In the data pre-processing stage of the experiment, data files in different formats are first read into memory to prepare for subsequent analysis. After reading, data filtering was performed based on the key labels for the different datasets. For the five datasets, including ETT, weather, and exchange rate, the data are filtered by the OT label. This filtering enables the precise extraction of core data, eliminates interference, and improves the analysis efficiency. The filtered data is converted into Python (3.10.15) lists. To enhance the computational efficiency, especially for large-scale numerical computations, the lists are converted into NumPy arrays. NumPy arrays offer efficient storage and fast computation, guaranteeing subsequent complex calculations. Using OT as the label, the data converted to NumPy arrays undergoes further processing. First, the mean and standard deviation of each feature are calculated to understand the data distribution characteristics, providing a reference for subsequent processing. Subsequently, normalization is performed using StandardScaler for Z-score standardization, transforming the data into a distribution with a mean of 0 and a standard deviation of 1. Although Z-score standardization theoretically has no upper or lower bounds for the data range, the data are concentrated within the interval [−3, 3], which allows for more reasonable weight allocation of each feature during model training and improves model performance. A fully overlapping sliding window was used to convert the time-series data into a format suitable for the model input, with a sliding step size of 1, which means that there are window_size-1 overlapping data points between adjacent windows. The sliding window is set to 60 h, with a size of 60 for the ETTh1 and ETTh2 datasets. Since the ETTm2 dataset has a 15-min sampling interval, its window size is 240. The window size for the weather dataset is set to 144, while that for the exchange_rates dataset is 7 (corresponding to a weekly period). By sliding a fixed-size window along the data, the forward and backward dependency relationships in the data are fully exploited to provide diverse training samples for the model. The dataset is divided into training and test sets at a ratio of 0.9:0.1. Finally, both the dataset and labels were converted into the PyTorch (2.1.1) tensor format. Finally, a direct single-step prediction strategy was adopted, implementing time-series prediction through a sliding window combined with the single-step prediction method.

### 4.5. Experimental Results and Analysis

The newly constructed TSEBG model was compared with popular deep learning models, including CNN-LSTM, LSTM [33], BiLSTM [34], GRU, TCN, and TransformerAttn. We first evaluated the performance of different models with varying window sizes on these three datasets by comparing the Root Mean Squared Error (RMSE), Mean Absolute Error (MAE), and Coefficient of Determination R^2^ on the final test set. The results are presented in Table 3 and Table 4.

On the ETTh1 datasets, TSEBG comprehensively outperforms all comparative models with an RMSE of 0.076, an MAE of 0.052, and an R^2^ of 0.924. Specifically, compared to the second-best LSTM model (RMSE 0.078), its prediction error is reduced by 2.6%, and the coefficient of determination R^2^ increases by 0.003, indicating a more stable fitting capability. Notably, in the ETTh2 datasets, although the GRU model achieves similar results in RMSE (0.108→0.080) and MAE (0.086→0.059) metrics, TSEBG’s R^2^ value (0.976) is still significantly 2.1% higher than that of GRU (0.956). This reveals that TSEBG can better capture the nonlinear features of the datasets, especially by maintaining a higher explanatory power under complex fluctuation patterns. On the ETTm2 dataset, its RMSE (0.021) and MAE (0.015) are 12.5% and 16.7% lower than those of TCN-LSTM, respectively. Meanwhile, the generally higher R^2^ may be attributed to the minute-level fluctuations and gentle data changes in this dataset. In terms of cross-dataset consistency, the standard deviation of R^2^ for TSEBG across the three datasets is only 0.037, which is significantly lower than that of the comparative models (such as 0.041 for GRU), demonstrating its stronger generalization ability. Notably, when handling datasets with obvious periodic mutations, like ETTh2 (where its MAE is 19.2% better than that of the second-best model). In the Weather prediction task, the TSEBG model demonstrates significant advantages. The experimental results show that our model has an excellent ability to interpret and fit weather data, far surpassing other models (e.g., the R^2^ values of CNN-LSTM and Transformer are 0.981, and that of GRU is 0.991, etc.), indicating a more precise capture of weather sequence patterns. On the Exchange_rate dataset, the TSEBG model has lower prediction errors than CNN-LSTM (RMSE 0.067, MAE 0.053), BiLSTM (RMSE 0.071, MAE 0.057), etc., enabling a more accurate prediction of exchange rate fluctuations. In both time-series prediction tasks of Weather and Exchange rate, TSEBG exhibits superior error control and data fitting effects, verifying its effectiveness and superiority in multi-scenario time-series prediction, with more prominent prediction accuracy and sequence pattern capture capability. TSEBG is comprehensively superior to the novel transformer framework in terms of prediction accuracy and fitting effect. It has a stronger adaptability and prediction ability for different types of time-series data, such as electricity, weather, and exchange rates. The results on these five datasets collectively demonstrate that the proposed model can adaptively balance the requirements of local feature extraction and global temporal modeling.

**Figure 4:**
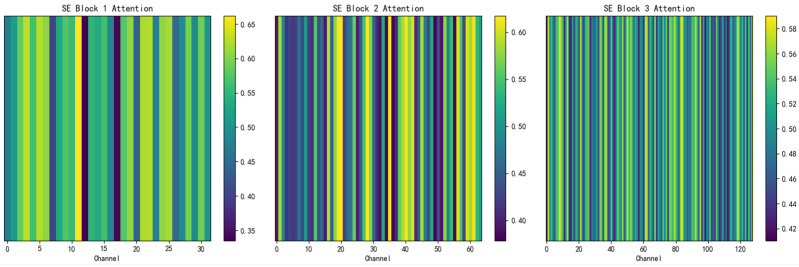
Visualization of the channel attention mechanism.

**Figure 5:**
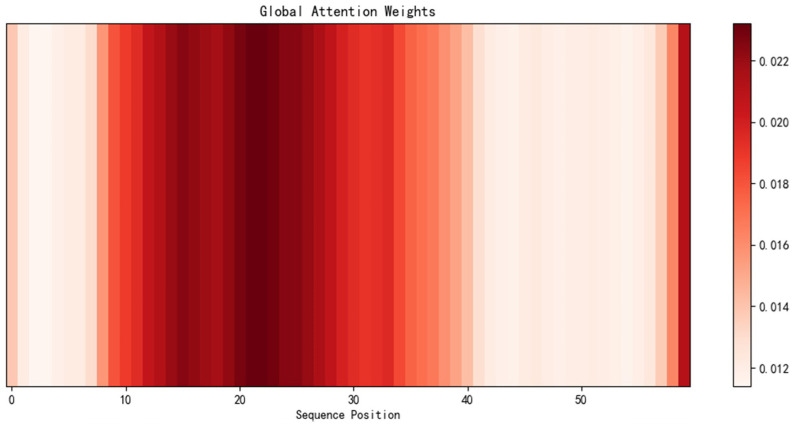
Visualization of the global attention mechanism.

The visualization of the attention mechanism for the TSEBG model is shown in Figure 4 and Figure 5.

**Figure 6:**
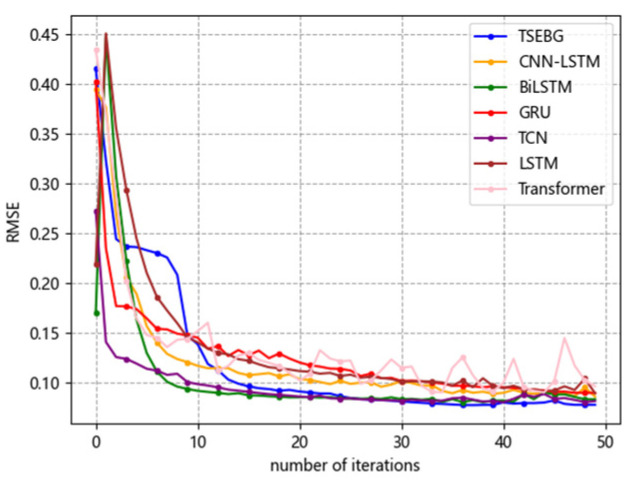
RMSE distribution (ETTh1).

**Figure 7:**
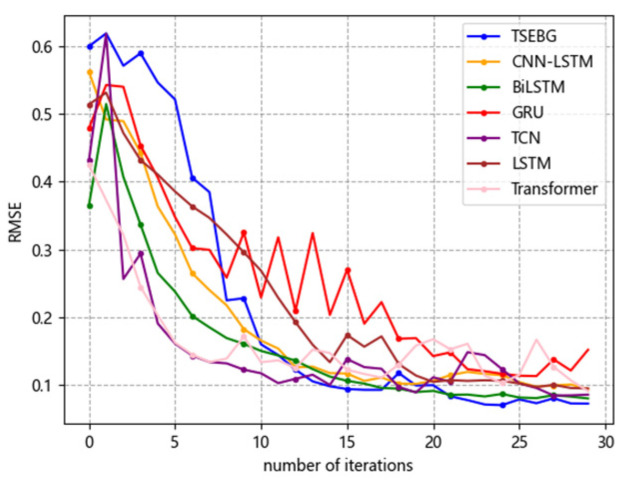
RMSE distribution (ETTh2).

**Figure 8:**
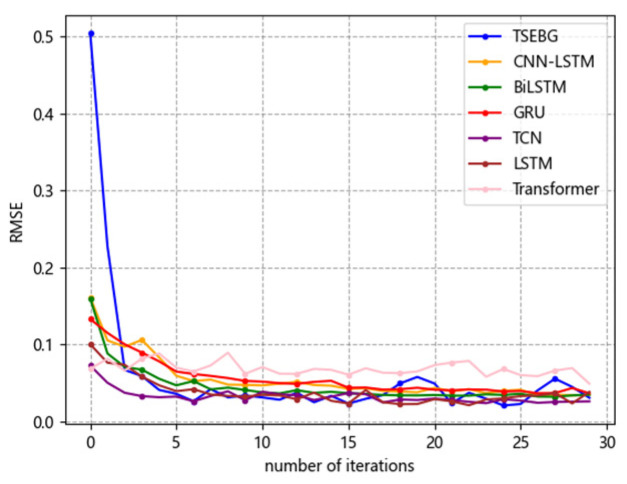
RMSE distribution (ETTm2).

**Figure 9:**
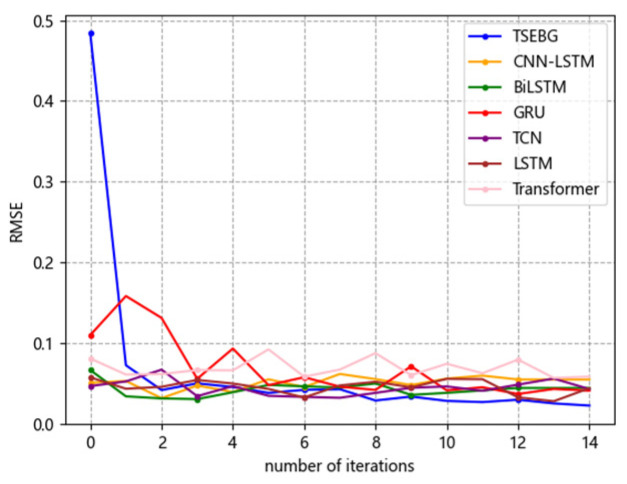
RMSE distribution (weather).

**Figure 10:**
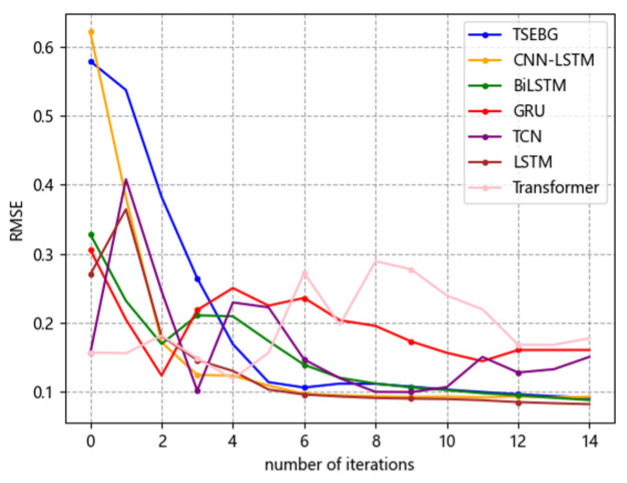
RMSE distribution (exchange_rate).

Figure 6, Figure 7, Figure 8, Figure 9 and Figure 10 show the RMSE distributions of the proposed model and several mainstream deep learning models in the test set. In these distributions, it is evident that our model has smaller RMSE values than the other models in time-series forecasting tasks.

**Figure 11:**
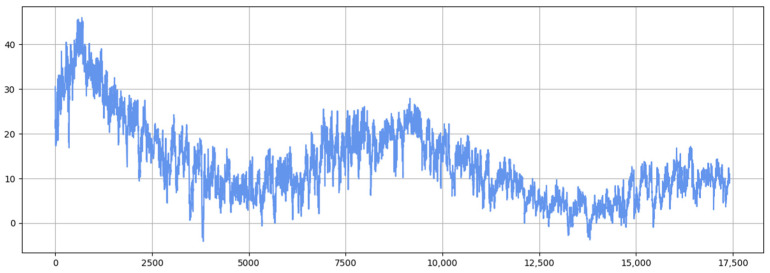
ETTh1 datasets.

**Figure 12:**
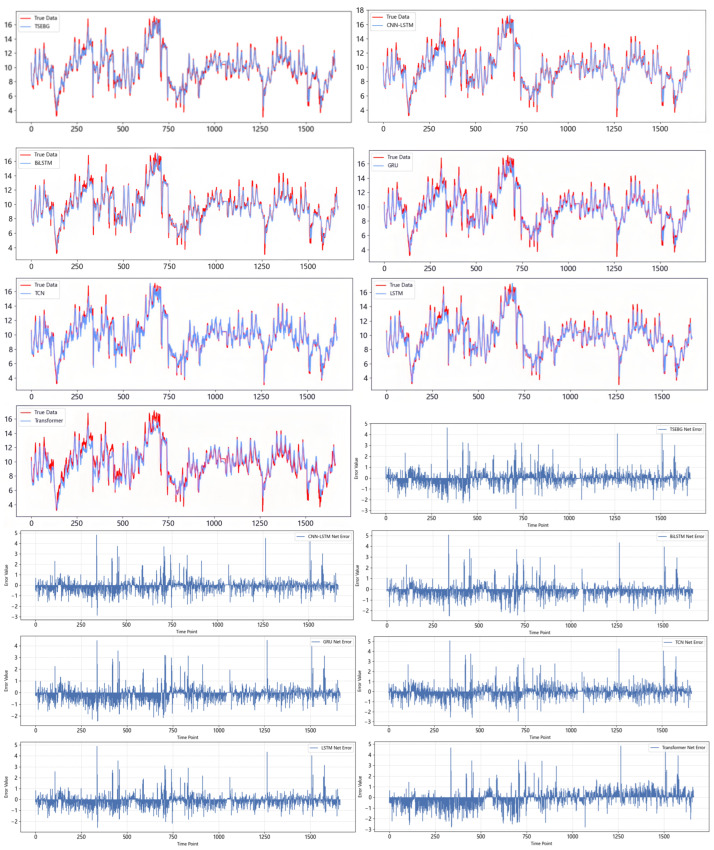
Comparison between predicted and actual values and error distribution on the ETTh1 dataset.

**Figure 13:**
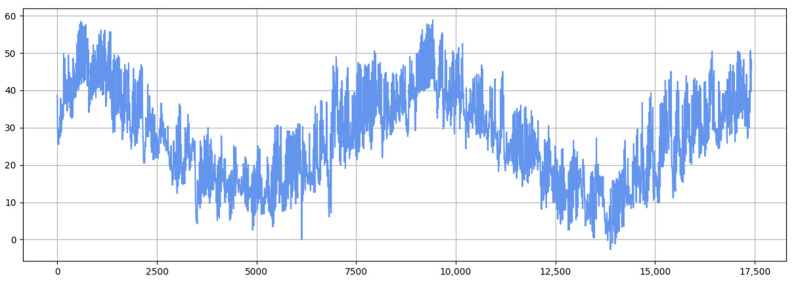
ETTh2 datasets.

**Figure 14:**
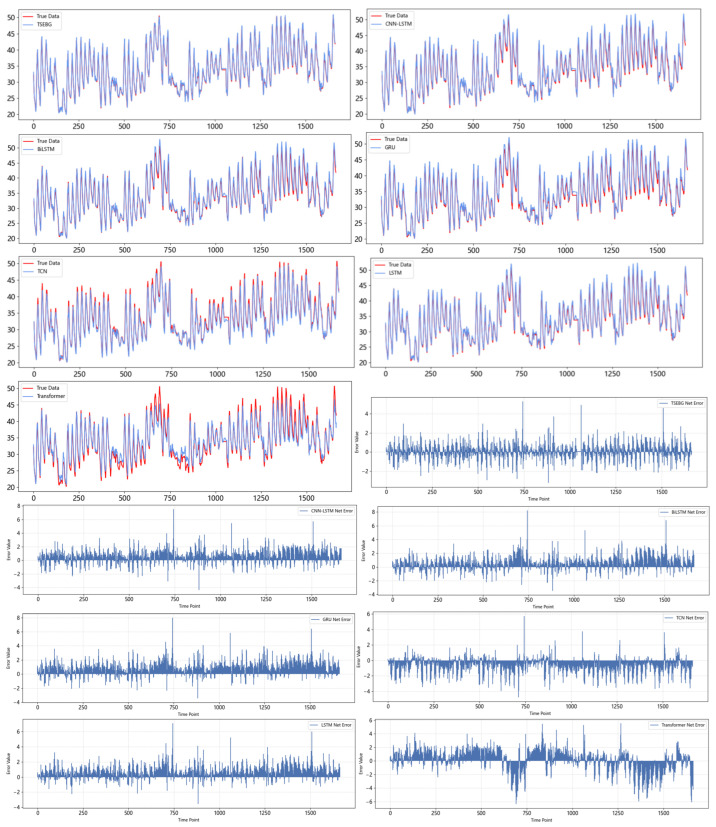
Comparison between predicted and actual values and error distribution on the ETTh2 dataset.

**Figure 15:**
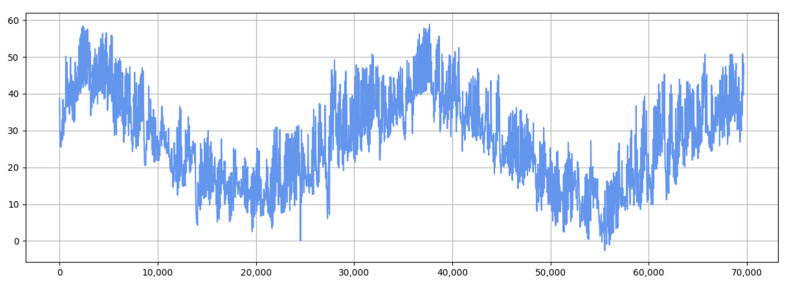
ETTm2 datasets.

**Figure 16:**
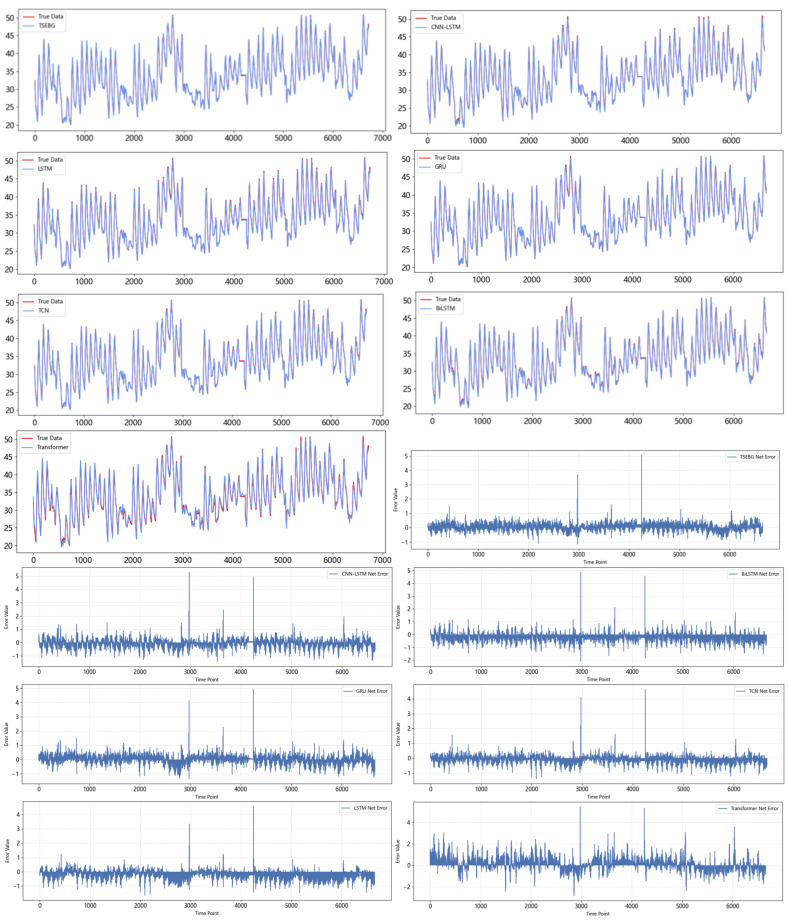
Comparison between predicted and actual values and error distribution on the ETTm2 dataset.

**Figure 17:**
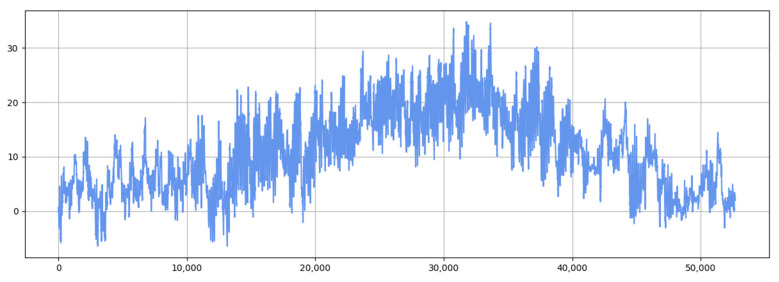
Weather datasets.

**Figure 18:**
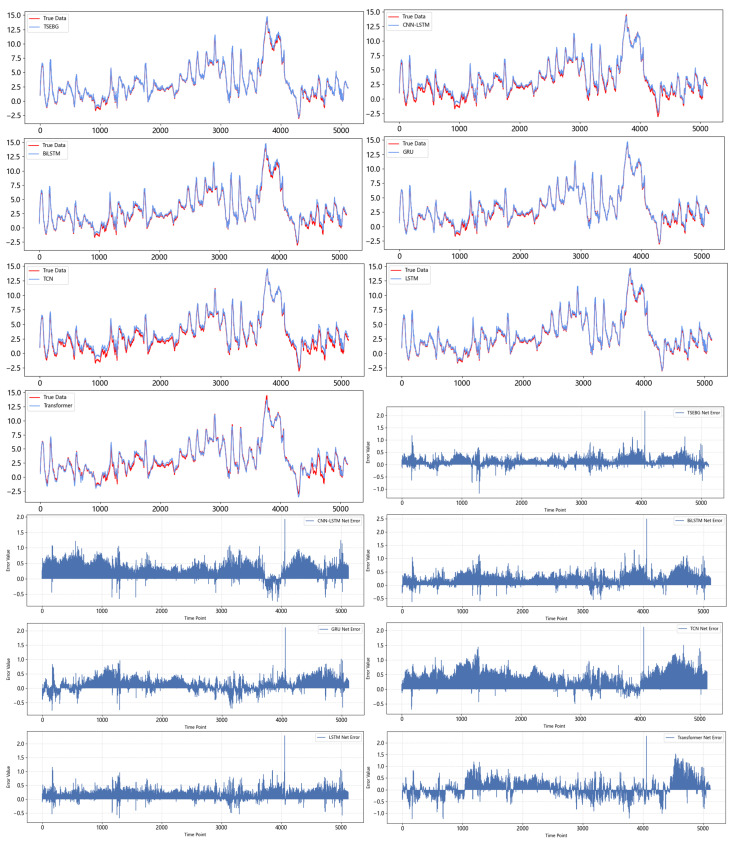
Comparison between predicted and actual values and error distribution on the weather dataset.

**Figure 19:**
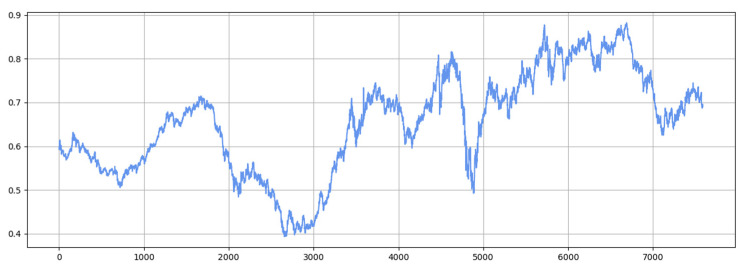
Exchange rate dataset.

**Figure 20:**
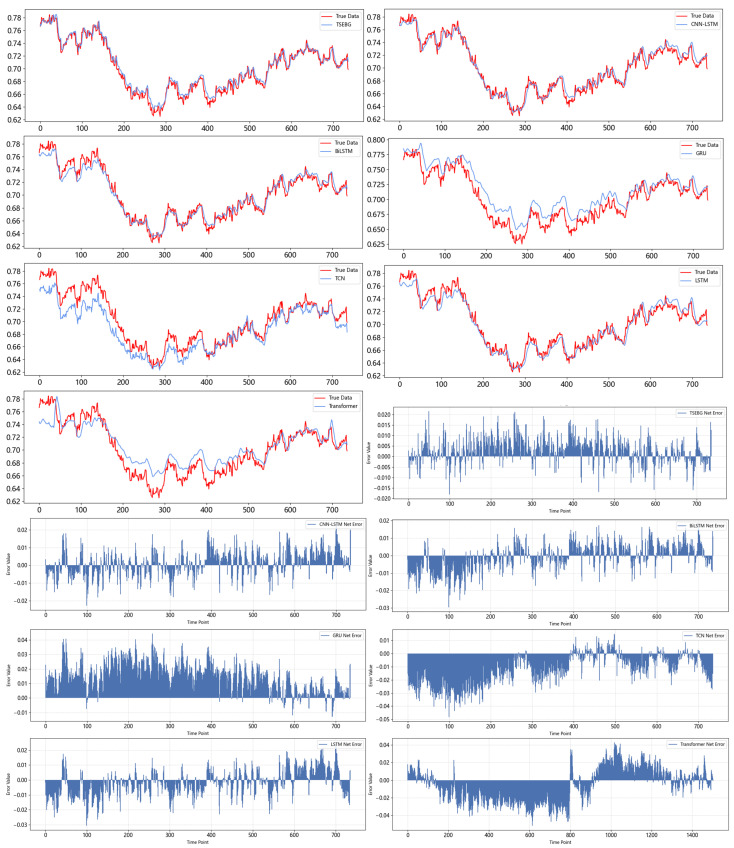
Comparison bBetween predicted and actual values and error distribution on the exchange rate dataset.

Figure 11 and Figure 12 show the visualization of the ETTh1 dataset and the comparison chart of test results between the TSEBG model and benchmark models on the ETTh1 dataset, with actual values provided. Figure 13 and Figure 14 present the visualization of the ETTh2 dataset and the visual results of prediction errors between our model and comparative models on the ETTh2 dataset. Figure 15, Figure 16, Figure 17, Figure 18, Figure 19 and Figure 20 respectively show the visualizations of the ETTm2, weather, and exchange_rate datasets, as well as the visual results of prediction errors between our model and comparative models on each dataset. In addition, we present the prediction error distribution of our model. From these comparative images, it can be intuitively seen that our model demonstrates a relatively stronger prediction ability on the test set.

### 4.6. Ablation Experiment

I will refine the operation process of the ablation experiments, emphasize the differences between different model combinations, and highlight the rigor and comprehensiveness of the experiments to enrich the content. When conducting in-depth research on the innovative TSEBG model, to accurately analyze the specific roles of each module in the model and their impact on model performance, we first constructed the TBG model by removing the SENet channel attention mechanism while retaining other modules. The SENet channel attention mechanism can adaptively adjust the weights between the channels. By removing this mechanism, we observed changes in the feature extraction and information fusion capabilities of our model. Next, we removed both the TCN module and the SENet channel attention mechanism to form the single-channel BG model, which retains only the BiGRU and GlobalAttention mechanisms. Finally, we constructed the TSEB and TSE models by removing the GlobalAttention mechanism and BiGRU-GlobalAttention component, respectively, to analyze the characteristics of each module in processing time-series data. The experimental results are shown in Table 5.

From the table above, we can see that the TBG model achieves an RMSE of 0.0772, MAE of 0.0531, and R^2^ of 0.918. Compared with the original TSEBG model, these accuracy metrics show a slight decline, indicating that the SENet channel attention mechanism plays a role in improving the model accuracy. After removing this mechanism, the model’s ability to capture the data features was slightly weakened. The accuracy metrics of the BG model were between those of the original and TBG models. With a training time of 853 s, removing the TCN module and SENet channel attention mechanism simplified the model structure, reducing the training time to some extent while causing relatively limited accuracy loss.

The TSE model had an RMSE of 0.0789, MAE of 0.0547, and R^2^ of 0.918, indicating relatively poor accuracy metrics. This demonstrates that the BiGRU and GlobalAttention mechanisms are crucial for capturing temporal dependencies and important features in time-series data; their removal significantly degrades the model performance. With a training time of 557 s, the shortest among all models, this indicates that the BiGRU and GlobalAttention mechanisms introduce a substantial computational complexity and training overhead. Removing these features simplifies the model structure and drastically reduces the training time.

## 5. Conclusions

Time-series data are widely distributed in fields such as finance, meteorology, and industry. Building predictive models that can accurately characterize the dynamic patterns and trends of data has become a current research focus. The TSEBG hybrid network model proposed in this study effectively addresses the limitations of traditional forecasting methods and some deep learning models in handling complex variable data. Given the challenge of fragmented processing of local and global information in traditional models, the TSEBG model constructs a hierarchical collaborative feature fusion network. Based on a dual-channel structure, it realizes complementary learning of multi-time-scale features and designs a cross-layer fusion strategy to achieve multi-level alignment of spatial-temporal information, significantly enhancing the representation capability of complex time-series data. The experimental results demonstrate that compared with mainstream deep learning models, the proposed model achieves an average reduction of 18.4% in RMSE and 21.9% in MAE, along with a significant improvement in R^2^, showcasing superior forecasting performance. It not only accurately captures short-term fluctuations but also effectively identifies long-term trends, providing a reliable solution for time-series data prediction.

Although this study achieved results, there is still room for improvement in model training. The model involves multiple groups of hyperparameters, such as TCN, BiGRU layers, and attention mechanisms, whose settings are interrelated and collectively determine the model’s performance. Hyperparameter tuning requires extensive experimental verification because each adjustment necessitates retraining and re-evaluation, which consumes time and effort. This poses challenges to researchers’ professional literacy and patience, further increasing the difficulty of optimizing the model. In practical application scenarios with high real-time requirements, it is necessary to comprehensively consider the model characteristics, balance the accuracy, resource consumption, and real-time performance, and perform optimization and verification. In future research, we plan to further optimize the model by introducing deep learning architectures, such as transformers. The self-attention mechanism of the transformer has significant advantages in processing long sequences and capturing global dependency relationships. We will explore the performance of different architectures in time-series prediction and screen the most suitable architecture to enhance model adaptability and performance, thereby promoting the development of time-series prediction research.

## Figures and Tables

**Table 1:** Datasets.

Database Name	Data Size	Adopt a Time Interval	Variables
ETTh1	17,421	1 h	7
ETTh2	17,420	1 h	7
ETTm2	69,681	15 min	7
Weather	52,696	10 min	21
Exchange_rate	7588	1 day	8

**Table 2:** Hyperparameters of the TSEBG model for different datasets.

Type	ETTh1	ETTh2	ETTm2	Weather	Exchange_Rate
Number of temporal blocks in TCN	3	3	3	3	2
The output dimension of each temporal block in TCN	32	32	64	64	32
	64	64	128	128	64
	128	128	64	64	-
Number of layers in BiGRU	2	2	2	2	1
The dimensionality of each layer in BiGRU	32	32	128	64	32
	64	64	64	32	-
Dimension of the attention layer	64	64	64	32	32
Convolution kernel	3	3	7	3	3
Embed_dim	128	128	256	64	128
Dropout	0.5	0.5	0.35	0.2	0.1

**Table 3:** The comparison results of different models on the ETT datasets.

Time-Series Model	ETTh1	ETTh2	ETTm2
	RMSE MAE R^2^	RMSE MAE R^2^	RMSE MAE R^2^
TSEBG	**0.077 0.053 0.922**	**0.070 0.053 0.981**	**0.022 0.016 0.998**
CNN-LSTM	0.079 0.055 0.918	0.101 0.079 0.962	0.031 0.023 0.996
BiLSTM	0.083 0.058 0.910	0.099 0.074 0.963	0.037 0.030 0.995
GRU	0.082 0.059 0.911	0.108 0.086 0.956	0.028 0.021 0.997
TCN	0.081 0.056 0.914	0.099 0.075 0.963	0.024 0.018 0.998
LSTM	0.078 0.055 0.921	0.091 0.069 0.969	0.034 0.027 0.996
TransformerAttn	0.101 0.076 0.866	0.092 0.069 0.900	0.062 0.047 0.986

**Table 4:** The comparison results of different models on the weather and exchange_rate dataset.

Time-Series Model	Weather	Exchange_Rate
	RMSE MAE R^2^	RMSE MAE R^2^
TSEBG	**0.031 0.024 0.994**	**0.060 0.048 0.968**
CNN-LSTM	0.055 0.048 0.981	0.067 0.053 0.962
BiLSTM	0.044 0.036 0.988	0.071 0.057 0.956
GRU	0.038 0.030 0.991	0.161 0.139 0.776
TCN	0.067 0.059 0.971	0.152 0.122 0.801
LSTM	0.035 0.030 0.992	0.084 0.068 0.939
TransformerAttn	0.055 0.042 0.981	0.178 0.150 0.917

**Table 5:** Ablation experiments on the ETTh1 dataset.

Time-Series Model	RMSE	MAE	R^2^	Training Time	Min MSE
TSEBG	0.0771	0.0527	0.922	885	0.0059
TBG	0.0772	0.0531	0.918	724	0.0059
BG	0.0780	0.0542	0.920	853	0.0058
TSEB	0.0855	0.0581	0.904	893	0.0073
TSE	0.0789	0.0547	0.918	557	0.0060

## Data Availability

The data presented in this study and the source code of the model can be obtained at https://github.com/LJL-WND/Time-series-forecasting-TSEBG.git (accessed on 20 June 2025).

## Appendix A

Table A1 lists the hyperparameter settings of comparison methods.

**Table A1:** Hyperparameter settings of comparison methods.

Datasets	Type	CNN-LSTM	BiLSTM	GRU	TCN	LSTM	Transformer
ETTh1	Hidden Layer	64	64	64	(32,64,128)	64	128
	Layer	2	3	3	3	2	3
	Kernel/Head	3			3		8
	Dropout	0.5	0.5	0.5	0.5	0.5	0.1
ETTh2	Hidden Layer	64	64	64	(32,64,128)	64	128
	Layer	2	3	3	3	2	3
	Kernel/Head	3			3		8
	Dropout	0.5	0.5	0.5	0.5	0.5	0.1
ETTm2	Hidden Layer	128	128	128	(64,128,64)	128	128
	Layer	3	3	3	3	2	2
	Kernel/Head	7			7		4
	Dropout	0.35	0.5	0.5	0.35	0.2	0.2
Weather	Hidden Layer	64	64	64	(64,128,64)	64	128
	Layer	3	3	3	3	2	2
	Kernel/Head	3			3		4
	Dropout	0.2	0.2	0.2	0.2	0.2	0.2
Exchange	Hidden Layer	64	64	64	(64,128,64)	64	64
	Layer	2	3	2	3	2	2
	Kernel/Head	3			3		4
	Dropout	0.1	0.1	0.2	0.2	0.2	0.2

## References

[1] Zhang Q., Qin C., Zhang Y., Bao F., Zhang C., Liu P. (2022). Transformer-Based Attention Network for Stock Movement Prediction. Expert Syst. Appl..

[2] Yao H., Wu F., Ke J., Tang X., Jia Y., Lu S., Gong P., Ye J., Li Z. (2022). Deep Multi-View Spatial-Temporal Network for Taxi Demand Prediction. Proc. AAAI Conf. Artif. Intell..

[3] Dong Y., Xiao L., Wang J., Wang J. (2023). A Time Series Attention Mechanism Based Model for Tourism Demand Forecasting. Inf. Sci..

[4] McLeod A.I., Li W.K. (1983). Diagnostic checking arma time series models using squared-residual autocorrelations. J. Time Ser. Anal..

[5] Torres J.L., García A., De Blas M., De Francisco A. (2005). Forecast of Hourly Average Wind Speed with ARMA Models in Navarre (Spain). Sol. Energy.

[6] Choi B. (1992). ARMA Model Identification.

[7] Anderson O.D., Box G.E.P., Jenkins G.M. (1978). Time Series Analysis: Forecasting and Control. Statistician.

[8] Hewamalage H., Bergmeir C., Bandara K. (2021). Recurrent Neural Networks for Time Series Forecasting: Current Status and Future Directions. Int. J. Forecast..

[9] Aadhitya A., Rajapriya R., Vineetha R.S., Bagde A.M. (2023). Predicting Stock Market Time-Series Data Using CNN-LSTM Neural Network Model. arXiv.

[10] Chung J.-Y., Gulcehre C., Cho K., Bengio Y. (2014). Empirical Evaluation of Gated Recurrent Neural Networks on Sequence Modeling. arXiv.

[11] Bai S., Kolter J.Z., Koltun V. (2018). An Empirical Evaluation of Generic Convolutional and Recurrent Networks for Sequence Modeling. arXiv.

[12] Duan Y., Liu Y., Wang Y., Ren S., Wang Y. (2023). Improved BIGRU Model and Its Application in Stock Price Forecasting. Electronics.

[13] Hu J., Shen L., Albanie S., Sun G., Wu E. (2020). Squeeze-and-Excitation Networks. IEEE Trans. Pattern Anal. Mach. Intell..

[14] Luong T., Pham H., Manning C.D. Effective Approaches to Attention-Based Neural Machine Translation. Proceedings of the 2015 Conference on Empirical Methods in Natural Language Processing.

[15] Peter Ď., Silvia P. ARIMA vs. ARIMAX–Which Approach is Better to Analyze and Forecast Macroeconomic Time Series. Proceedings of the 30th International Conference on Mathematical Methods in Economics.

[16] Vogels T.P., Rajan K., Abbott L.F. (2005). Neural network dynamics. Annu. Rev. Neurosci..

[17] Thissen U., van Brakel R., de Weijer A.P., Melssen W.J., Buydens L.M.C. (2003). Using Support Vector Machines for Time Series Prediction. Chemom. Intell. Lab. Syst..

[18] Gui B., Wei X., Shen Q., Qi J., Guo L. Financial Time Series Forecasting Using Support Vector Machine. Proceedings of the 2014 Tenth International Conference on Computational Intelligence and Security.

[19] Wang H., Hu D. Comparison of SVM and LS-SVM for Regression. Proceedings of the 2005 International Conference on Neural Networks and Brain.

[20] Sun A., Zhao T., Chen J., Chang J. (2018). Comparative Study: Common ANN and LS-SVM Exchange Rate Performance Prediction. Chin. J. Electron..

[21] Zhao Z., Xia C., Chi L., Chang X., Li W., Yang T., Zomaya A.Y. (2021). Short-Term Load Forecasting Based on the Transformer Model. Information.

[22] Wu H., Xu J., Wang J., Long M. (2021). Autoformer: Decomposition Transformers with Auto-Correlation for Long-Term Series Forecasting. arXiv.

[23] Liu Y., Wu H., Wang J., Long M. (2022). Non-Stationary Transformers: Rethinking the Stationarity in Time Series Forecasting. arXiv.

[24] Ullah K., Ahsan M., Hasanat S.M., Haris M., Yousaf H., Raza S.F., Tandon R., Abid S., Ullah Z. (2024). Short—Term load forecasting: A comprehensive review and simulation study with CNN—LSTM hybrids approach. IEEE Access.

[25] Dasgupta S., Osogami T. (2022). Nonlinear Dynamic Boltzmann Machines for Time-Series Prediction. Proc. AAAI Conf. Artif. Intell..

[26] Wen X., Li W. (2023). Time Series Prediction Based on LSTM-Attention-LSTM Model. IEEE Access.

[27] Cai J., Zhang K., Jiang H. (2023). Power Quality Disturbance Classification Based on Parallel Fusion of CNN and GRU. Energies.

[28] Sherstinsky A. (2020). Fundamentals of Recurrent Neural Network (RNN) and Long Short-Term Memory (LSTM) Network. Phys. D Nonlinear Phenom..

[29] Arumugham V., Ghanimi H.M.A., Pustokhin D.A., Pustokhina I.V., Ponnam V.S., Alharbi M., Krishnamoorthy P., Sengan S. (2023). An Artificial-Intelligence-Based Renewable Energy Prediction Program for Demand-Side Management in Smart Grids. Sustainability.

[30] He K., Zhang X., Ren S., Sun J. (2016). Identity Mappings in Deep Residual Networks. Computer Vision—ECCV 2016.

[31] Yang Y., Zhong J., Li W., Gulliver T.A., Li S. (2020). Semisupervised Multilabel Deep Learning Based Nonintrusive Load Monitoring in Smart Grids. IEEE Trans. Ind. Inform..

[32] Zhu Q., Zhang F., Liu S., Wu Y., Wang L. (2019). A Hybrid VMD–BiGRU Model for Rubber Futures Time Series Forecasting. Appl. Soft Comput..

[33] Kharel A., Arean Z., Kaur D. (2024). Long Short—Term Memory (LSTM) Based Deep Learning Models for Predicting Univariate Time Series Data. Int. J. Mach. Learn..

[34] Wang H., Zhang Y., Liang J., Liu L. (2023). DAFA-BiLSTM: Deep Autoregression Feature Augmented Bidirectional LSTM Network for Time Series Prediction. Neural Netw..

